# The Effects of Priming Negative Cues on Stroop Rifle Shooting Task in Elite Biathletes: A Psychophysiological Pilot Study

**DOI:** 10.1177/00315125261419408

**Published:** 2026-01-28

**Authors:** Khelifa Bartura, Henrik Gustafsson, Frank Eirik Abrahamsen

**Affiliations:** 1 Department of Sport and Social Sciences, 570071 Norwegian School of Sport Sciences, Oslo, Norway; 2 National Research Center for Youth Sports, Department of Educational Studies–Sport Sciences, Karlstad University, Sweden

**Keywords:** cue frequency, ironic error, HRV, priming negative cues, rifle shooting, Stroop task

## Abstract

Attempting to suppress unwanted thoughts is generally characterized by the opposite outcomes of what is desired in motor task performance, resulting in a counterintentional error. The study examined the impacts of priming negative cues on elite athletes’ performance in Stroop rifle shooting task, guided by ironic processes of mental control theory (Wegner, 1994). Ten elite youth biathletes performed the task under two conditions: Low-Cue Frequency (LCF) and High-Cue Frequency (HCF) using a quasi-experimental within-subject design. Using multiple psychophysiological measures, the study assessed ironic errors, non-ironic errors, target hits, and reaction times (RTs) at the incongruent stimuli. Across both conditions, repeated negative priming cues did not lead to ironic shooting errors or delays in target responses—contradicting Wegner’s (1994) assumption about ironic errors. Bayesian analysis revealed moderate and anecdotal evidence in support of the null model (BF_01_) for target hit rates and RTs towards target hits, respectively. Heart rate (HR) and heart rate variability (HRV) also showed no significant variations across conditions. Findings suggest that negative instructions, regardless of frequency, do not disrupt elite biathletes’ shooting performance. Directives like “do not shoot [specific color]” fail to induce ironic errors, highlighting elite biathletes’ resilience to such cues in maintaining consistent shooting performance.

## Introduction

Many athletes often experience unwanted pre-performance thoughts, such as trying not to miss a decisive shot. For instance, just before the penalty shootout, a football player said, “You don’t want to ruin it all. You don’t want to not go through to the next round because YOU missed. So, you just hope that you will not miss your penalty. And you think: Don’t miss, don’t miss, don’t miss, DON'T MISS! That’s all I’m thinking” (Jordet, 2024, p. 40). Athletes occasionally perform in a manner that is precisely opposite to their intentions. This phenomenon has been investigated in the sport and performance psychology literature over the past two decades, particularly when athletes intentionally trying to suppress unwanted thoughts.

Within the ironic processes of mental control theory (Wegner, 1994; hereafter *IPT*), processing unwanted thoughts has been characterized by *counterintentional effect*, where an individual shows an opposite behavior of what is desired (Wegner, 1994). Central to IPT lies the foundational idea that mental control is governed by the interaction of two hypothetical yet competing cognitive processes: an intentional (conscious) operating process and an ironic (unconscious) monitoring process. The operating process is deliberate, effortful, and consciously directed, enabling individuals to pursue and achieve the desired mental states. In contrast, the monitoring process operates automatically, effortlessly, and outside conscious awareness, scanning for thoughts or stimuli that could undermine those desired states (Wegner, 1994). Both processes stem from the same control system and operate together in a feedback loop, keeping unwanted thoughts out of conscious awareness and maintaining effective mental control (Wegner, 1994).

When individuals attempt to suppress an unwanted thought, they not only fail to do so but ironically find themselves dwelling on the very thought they are trying to avoid (Wegner, 2009). Wegner (1994) termed this phenomenon as *ironic effect* because the monitoring process, designed to detect control failures, can cause them. This effect is ironic as suppressing unwanted thoughts, intended to reduce their prevalence and accessibility, instead the monitoring process makes them more prominent and accessible to the conscious mind (Wegner, 1994). Similarly, the effort to control an intended or ongoing motor action (such as “do not miss the shot”) may ironically increase the mental activation of “missing,” thereby raising the probability that it will negatively affect motor execution (Wegner, 1994; Wegner et al., 1998).

The existing evidence has shown the detrimental effects of suppressing negative instructions under pressure, leading to ironic errors in motor performance (Bartura et al., 2023). However, a critical gap remains in understanding these effects using a *cognitive-motor dual-task paradigm*—a methodology central to this paper. For example, golfers instructed to avoid overputting while mentally rehearsing a number tended to overshoot the target (Wegner et al., 1998). Similarly, dancers instructed to avoid wobbling while counting numbers backward demonstrated a reduced balance (Dugdale & Eklund, 2003).

While mental arithmetic tasks can challenge working memory and attention but are often criticized for lacking ecological validity, as they fail to replicate the complex and dynamic nature of real-world sports environments (Woodman et al., 2015). Similarly, motor tasks in IPT research, such as balance task on a wobble board for dancers, often fail to reflect the specific movement skills relevant to actual performance context.

A key challenge in cognitive-motor dual-task studies examining IPT lies in defining ironic errors—errors resulting from suppressed thoughts under cognitive load. This is complicated by the difficulty of distinguishing these *specific errors* from *generic* performance lapses caused by stress or external pressures. For instance, in Wegner et al.’s (1998) golf putting experiment, over-hit putts were defined as ironic errors^1^, while under-hit putts were excluded, potentially conflating ironic errors with general performance declines. This highlights the need for clear operational definitions to separate ironic errors from non-ironic ones (Gorgulu et al., 2019), ensuring accurate empirical validation of IPT. Furthermore, while cognitive load is a crucial element for ironic errors, this phenomenon can occur irrespective of cognitive load. The classic “white bear” puzzle demonstrates this: when participants are instructed not to think of a white bear, they often think about it (Wegner et al., 1987, Experiment 2). This effect is not limited to thoughts; it also impact motor actions. In golf putting studies, nearly 21% of participants showed ironic error when instructed not to putt the ball short, irrespective of load (de la Pena et al., 2008, Study 1). A follow-up experiment confirmed this with 16.7% of participants making ironic error when instructed not to leave the ball short or long despite having no cognitive load at all (de la Pena et al., 2008, Study 2). This evidence suggests the real trigger for these errors is the negative instruction itself. The very act of trying to suppress a thought or action can paradoxically bring it to the forefront of our conscious mind, ultimately steering our behavior toward the exact mistake we are trying to avoid.

Another notable concern in studies examining IPT using the cognitive-motor dual-task method is the reliance on performance-based metrics (like reaction times, error rates) to assess cognitive load (e.g., Binsch et al., 2010; Wegner et al., 1998). While these measures are considered objective (Tenenbaum & Filho, 2018), they fail to capture the full scope of cognitive load effects, highlighting the need for a multi-measure approach that includes psychophysiological indices (Wilson et al., 2007). Previous studies using mental arithmetic in dual-task paradigms have not incorporated psychophysiological measures like heart rate (HR) and heart rate variability (HRV) to quantify cognitive load (for a review, see Bartura et al., 2023). Notably, HRV, an indicator of the link between parasympathetic and cardiac activity, providing an objective measure of athletes’ response to stress (Laborde et al., 2011) and emotion regulation (Thayer et al., 2009). According to Thayer et al.'s (2009) neurovisceral integration model, HRV reflects prefrontal-parasympathetic (vagal) interactions, linking the central autonomic network to cognitive processes like attention, inhibition, and working memory. Higher HRV signifies efficient physiological and behavioral adaptability, while lower HRV is associated with reduced flexibility and impaired performance in dynamic environments (Thayer et al., 2009). Thus, incorporating HRV could offer a comprehensive insight on how negative instructions influences ironic errors.

Research on IPT in motor task performance reveals challenges in identifying the cause of ironic performance errors (Bartura et al., 2023). Studies often use explicit instructions paired with priming phrases (e.g., ‘do not putt the ball short’) to guide participants behavior. However, it remains unclear whether the resulting errors stem from the instructions, priming effects or the increased accessibility of avoidant behavior (Woodman et al., 2015). Research in mainstream psychology indicates that the more frequently an unwanted thought is activated (or primed) by the automatic search mechanism, the more likely it is to enter conscious awareness, regardless of cognitive load, increasing unwanted effects (Macrae et al., 1994). In motor performance setting, Beilock et al. (2001) showed that repeatedly priming^2^ suppressive imagery, such as instructing participants not to image undershooting a target, resulted in overshooting during golf putting. Moreover, studies suggest that ironic errors occur when participants focus on repetitive negative instructions during simulated soccer penalty tasks (e.g., Bakker et al., 2006; Binsch et al., 2010). However, the influence of repeated priming negative instructions on ironic errors remains inconsistent, making the relationship between instructions and unwanted errors ambiguous.

Finally, a critical challenge in testing IPT is the debate over the prevalence of ironic errors among elite athletes. Early scholarly works dismissed such errors as “relatively infrequent” among elite athletes (Hall et al., 1999, p. 221; Janelle, 1999), with Woodman and Hardy (2001) noting their obviousness when they occur, while Woodman and Davis (2008) called them “rare events” (p. 192). However, empirical evidence showed that elite shooters made ironic errors under high-anxiety conditions (Gorgulu, 2019). Yet the prevalence of ironic errors in elite athletes remains unclear due to limited evidence, emphasizing the need for further research across diverse sports and conditions.

### The Present Study

This study aimed to address prior limitations by using a Stroop rifle shooting task with multiple psychophysiological measures to test IPT. Specifically, it investigated whether repetitive priming or activation of negative cues leads to ironic errors in elite biathletes regardless of cognitive load. The Stroop task, a foundational dual-task paradigm, measures cognitive changes such as attention and inhibition by requiring individuals to select and identify one dimension of a bidimensional stimulus (e.g., word meaning versus ink color) (MacLeod, 1991; Stroop, 1935). Its two common variants—classical (color identification) and reverse (word identification)—show slower response to incongruent stimuli (e.g., the word “RED” in blue ink) than congruent ones (e.g., the word “RED” printed in red).

This study used a rifle shooting task to examine how unwanted thoughts impact elite shooters’ performance. Evidence shows that when elite shooters become preoccupied with thoughts such as fear of mistakes or missing the target, it often results in missing their decisive shots (see Josefsson et al., 2019; Lindner, 2017). Negatively phrased instructions, from coaches or self-generated, increase sensitivity to such thoughts, causing performance breakdowns in motor skills such as shooting performance (Oudejans et al., 2013). Despite the importance of mental control in far-aiming motor performance (Payne et al., 2019), the psychological effects of unwanted thoughts during the pre-shot phase remain understudied.

Consequently, we hypothesized that under high-cue frequency conditions, biathletes would make more errors when instructed not to shoot a particular color (e.g., “Do not shoot blue”) due to the ironic processing of negative commands. We also expected slower reaction times for correct responses in incongruent trials under high-cue frequency compared low-cue frequency conditions. Specifically, we predicted that the “do not shoot [color]” instruction would impose significant cognitive demand (by introducing a complex, multi-stage cognitive process into shooting sequence) on biathletes before each shot, requiring them to: first, encode the instruction before a stimulus appeared; then, rapidly identify the target and distractor; compare the target’s color to the instruction; focus their attention accordingly; make a critical go/no-go decision; and finally, execute a precise shot or completely inhibit the prepared motor action.

## Method

### Participants

Thirteen elite^3^ biathletes, aged 16 to 18, from the Norwegian Elite Sport Academy (“Norges Toppidrettsgymnas,” NTG), Geilo participated in the study. Two withdrew due to injury and illness, and one was excluded because of a technical malfunction during video recording, resulting in a final sample of 10 elite youth biathletes (5 female; 3 left-handed) with a mean age of 16.8 (±0.92) years. Affiliated with various Norwegian biathlon clubs, the athletes averaged 7.4 (±1.96) years of training and 6.6 (±1.71) years of competition experience. All biathletes had normal color vision and reported no physical limitations affecting their rifle shooting performance.

#### Task

Based on Wood et al. (2016),^4^ the task used a reverse-Stroop design. Each stimulus featured a black circle with a centered word (“blue,” “green,” “yellow,” or “red” in Norwegian, Arial font size 40, 24 cm diameter) and four colored targets (blue, green, yellow, or red) in the corners, as shown in Figure 1. Each target consisted of an outer colored circle (30 cm diameter) and an inner white circle (12 cm diameter). The word and target colors were either congruent (e.g., “blue” in blue) or incongruent (e.g., “blue” in red).

**Figure 1.**
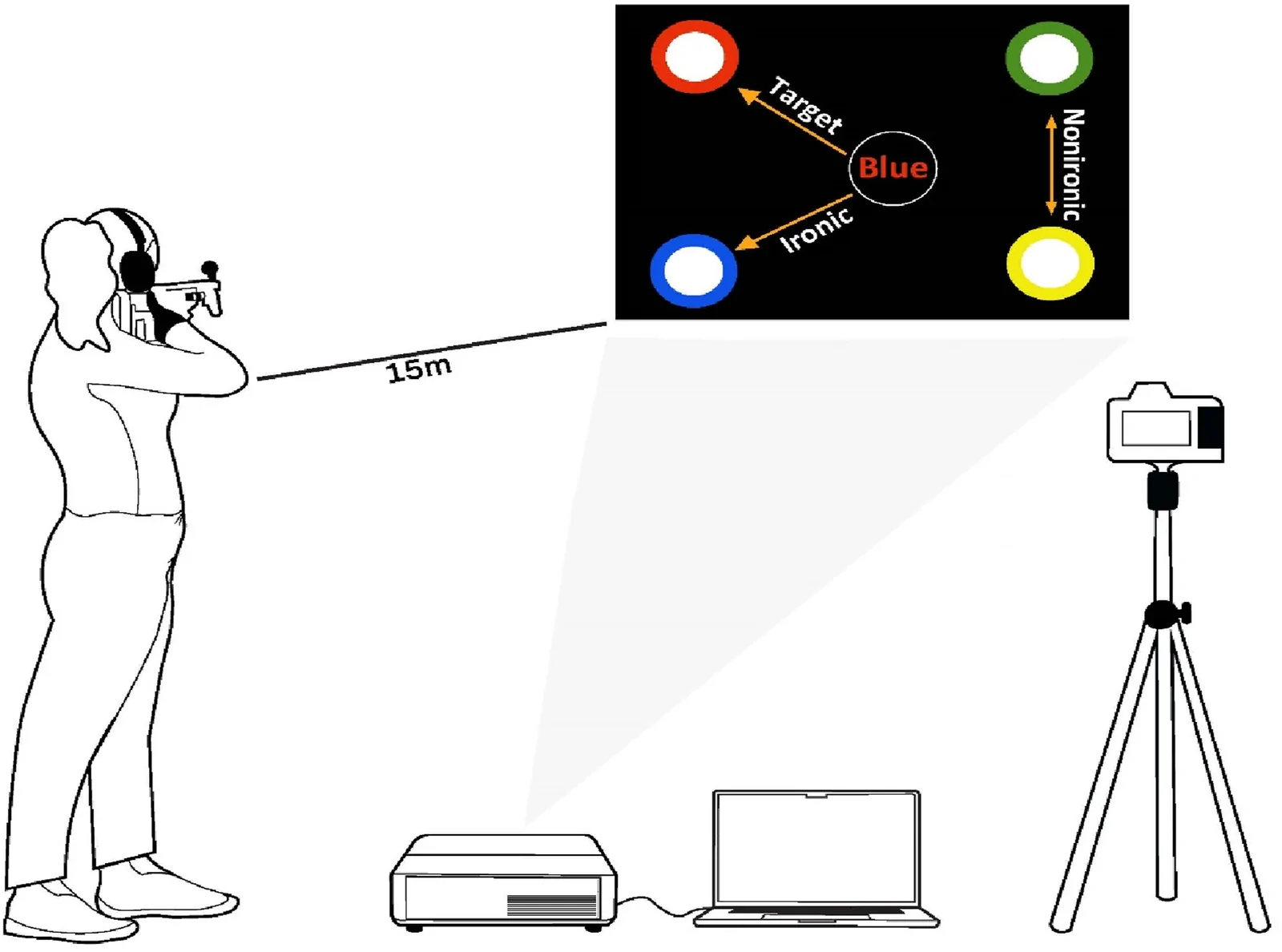
Incongruent stimulus showing conceptualization of target, ironic error target, and non-ironic (non-target) error target when given do not shoot [BLUE] instruction *Note*. As shown in Figure 1, a shot on the **RED** target is a correct **Target Hit** (with its white zone as the “bullseye”). A hit on the **BLUE** target is an **Ironic Error**, and any shot that lands elsewhere or a complete failure to fire is classified as a **Non-Ironic Error**. The incongruent stimulus is when the word “BLUE” is written in RED ink. The congruent stimulus, the word “BLUE” is written in BLUE ink

#### Task Conditions

The task involved two conditions: low-cue frequency condition (LCF) and high-cue frequency condition (HCF). The LCF condition presented a balanced proportion of congruent and incongruent stimuli (10 trials each). The HCF condition incorporated an unmixed block of 20 incongruent trials (following Neill, 1977). The target and word of each stimulus were counterbalanced across conditions.

#### Instructional Manipulation

Prior to the trial under LCF and HCF conditions, biathletes were given explicit task instructions verbally and using a graphical material on a secure Apple iPad 11Pro.

#### LCF Condition

Biathletes were told they would see a mix of congruent and incongruent color-word stimuli. They were instructed to pay particular attention not to shoot the target that matches the word’s meaning” for *incongruent trials* (e.g., “blue” in red) and “shoot the target whose color matches the word’s ink” for the *congruent trials* (e.g., “blue” in blue). Before their first shot, they were also verbally instructed to shoot “as quickly and accurately as possible” as each stimulus disappears in 2 seconds.

#### HCF Condition

Biathletes were informed that the next trial would consist only of incongruent stimuli. They were instructed to be especially careful to avoid shooting any target where the color corresponded to the word’s meaning. Before each stimulus, a negative instructional cue (e.g., “Do not shoot BLUE”) would flash on the screen. Biathletes were told to carefully adhere to these cues while shooting as quickly and accurately as possible. The time of stimulus presentation across conditions is detailed in Figure 2.

**Figure 2.**
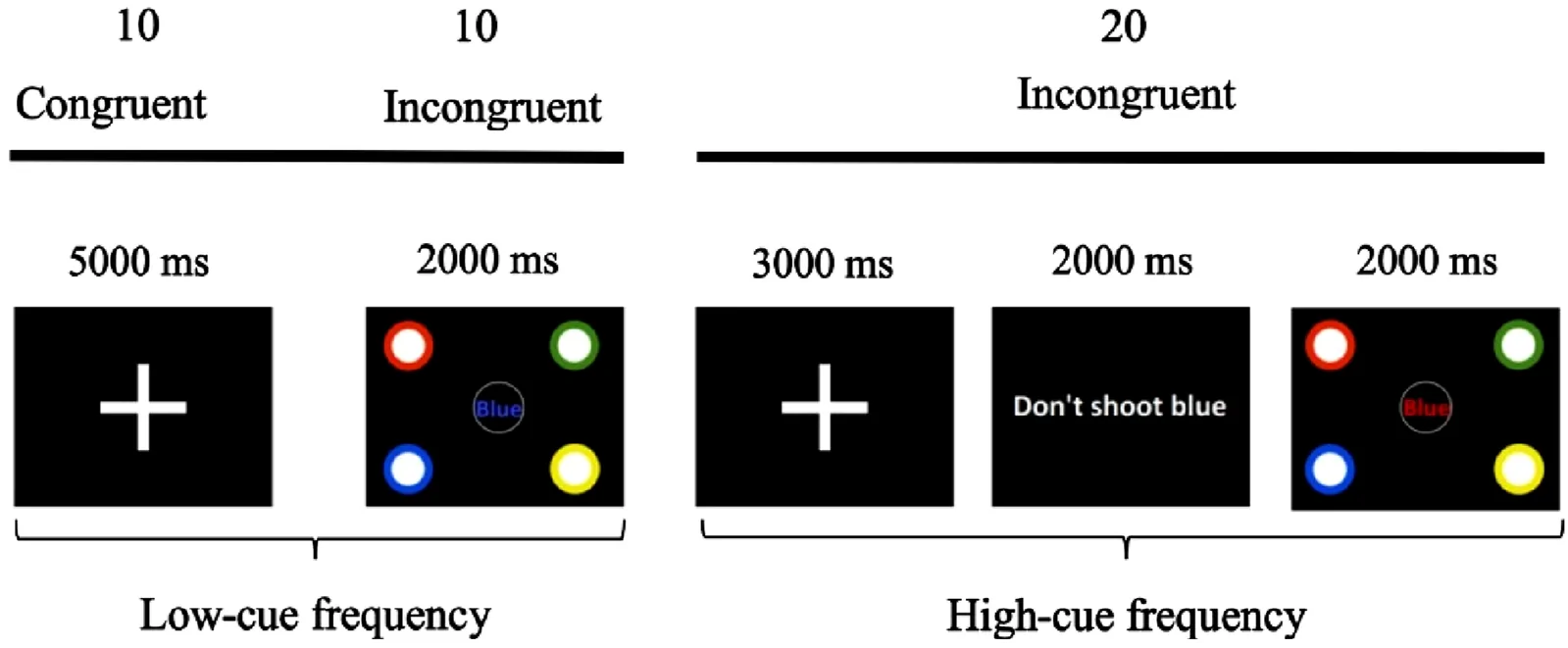
Presentation of stimulus and proportion of congruent and incongruent stimuli across trial conditions *Note*. **Stimulus timing for LCF condition*:*** Each target was preceded by a central white fixation cross (+), which was displayed 5000 milliseconds. **Stimulus timing for HCF condition*:*** The standard 5000 milliseconds pre-stimulus period was divided into two phases: the central fixation cross was shown for the first 3000 milliseconds and the negative cue was then visually displayed for the remaining 2000 milliseconds. Each target stimulus disappeared after 2000 milliseconds and a blank black slide was shown for 15,000 milliseconds to allow for magazine changes across conditions

## Measures and Materials

### Psychological Measures

To determine whether biathletes’ trait anxiety scoring influences their rating on state anxiety, we used the sport anxiety scale (SAS-N, Abrahamsen et al., 2006). Having negative (trait) appraisals before shooting targets is more common among elite shooters (Calmeiro et al., 2014). Cognitive anxiety was measured using the mental readiness form (MRF-3; Krane, 1994). The MRF-3 is a widely used subjective tool for studying the relationship between pressure and attention in far-aiming motor tasks (Payne et al., 2019).

The perceived workload during the shooting task was assessed using the mental demand, temporal demand, and effort subscales of the NASA-TLX (Hart & Staveland, 1988). This multidimensional tool is widely used in cognitive tasks, such as the Stroop test (e.g., Staiano et al., 2023) due to its ease of administration.

### Physiological Measures

HR and HRV were recorded using the polar H10 transmitter to measure biathletes’ physiological arousal in response to the instructional manipulation. HRV is commonly used in Stroop task research in sporting performance settings (e.g., Gantois et al., 2019; Sartor et al., 2017).

### Manipulation Checks

A pre-instruction manipulation check ensured biathletes understood the instructions. After the completion of the experiment, a Likert scale (1 = strongly disagree, 4 = strongly agree) measured their agreement with social evaluative statements.

### Color Blind Test

Biathletes were screened for red-green color vision deficiency with the Ishihara 24-plate test (Ishihara, 1972). A minimum score of 10 correct responses on 15 plates was required for inclusion.

### Performance Measures

The Stroop rifle shooting task measured shooting performance from a 15-m range. Performance outcomes were conceptualized based on the recommendations of Woodman et al. (2015) and Gorgulu et al. (2019). Particularly, in the Stroop shooting task, biathletes’ responses on incongruent trials, where the color word and the target color did not match, were classified into three distinct outcomes categories based on the instruction “do not shoot [specific color].” The first outcome was a correct Target hit, defined as hitting a target that matched the color of the distractor’s ink, such as hitting the RED target when the word “BLUE” was written in red). The second was an Ironic Error, which occurred when a biathlete hit the very target they were instructed to avoid; for example, hitting the BLUE target when the distractor word “BLUE” was printed in red ink. The third category was a Non-ironic (non-target) Error. This included hitting any task-irrelevant target (e.g., green or yellow one), as well as other performance failures like missing the target entirely, misfiring, or failing to fire within the allotted time limit (see Figure 1). The second performance measure was shooting RTs, defined as the latency between the presentation of the stimulus and the biathlete’s successful shot at the correct target. Faster RTs indicated efficient task processing, while slower RTs reflected the cognitive interference of incongruent trials.

### Apparatus

A secured laptop was connected to a projector, placed on a 12 cm-high surface, to display stimuli. A portable wooden frame canvas (190 × 100 cm) was constructed, covered with white paper, and securely attached to a standard indoor shooting range wall. The projector, positioned 240 cm from the canvas, projected a 160 × 90 cm image. From a viewing distance of 15 meters, the projected image subtends a visual angle of approximately 6.11^o^ horizontally and 3.44^o^ vertically. A new sheet of white paper was used for each trial as reusing the paper would make it impossible to assign bullet holes to the correct trial. Stimulus presentation was initiated using a wireless presenter.

A video camera was mounted on a 120 cm tripod, recording biathletes’ shooting marks on the canvas. The position of the camera was placed 3-m to the right side (∼30^°^) of the shooting range wall. This configuration safeguards the camera and offers an optimal oblique angle to precisely capture bullet impacts across the entire target area.

The biathletes used their personal rifles and clothing. Both the biathletes and the experimenters wore earmuffs to protect against noise from the gunshots. The specifications for the apparatus and software used in this study are provided in Supplemental Table 1.

### Testing Procedure

Biathletes completed a standing Stroop rifle shooting task in a single 30–45 minute session at the NTG Geilo’s indoor shooting range facility. Appointment times were scheduled based on their study and training timetables. Before testing, the research assistant collected consent, demographic data, baseline cardiac data, SAS-N scores, and color blindness results. Each biathlete was tested individually. Upon arrival, the main researchers explained the experiment’s details.

First, biathletes received a “do not shoot [specific color, e.g., blue]” instruction under LCF and HCF conditions. Verbal commands were used for LCF and visual commands for HCF. This was followed by a check for instruction comprehension and an administration of MRF-3. Biathletes then performed 20 shots under LCF and 20 under HCF conditions, with HR and HRV measured separately for each condition. Following each trial block, a 5-min break was provided. During this time, biathletes completed the NASA-TLX questionnaire and loaded their magazine for the subsequent trial block. The session ended with a post-test survey and a debriefing, where we thanked biathletes for their participation. Supplemental Figure 1 outlines the experimental protocol.

## Data Reduction

The shooting performance was analyzed frame-by-frame using Camtasia software. This involved recording hit locations (target, ironic error, or non-ironic error) and latency (in milliseconds) from stimulus display to bullet hit on the canvas. Durations associated with the presentation of the fixation stimulus, negative cues, and blank slide were excluded.

## Data Management

Data were managed with SPSS 28. Normality test results confirmed a normal distribution of the data, with no missing values or outliers identified. Biathletes’ cardiac data were recorded and exported using the Polar Equine application on a secured smartphone. The raw electrocardiography (ECG) data (HR.txt) were then converted into comma-separated values (HR.csv) format and imported into Kubios software for analysis. HRV was computed for epochs spanning from 5000 milliseconds pre-stimulus to either 4000 milliseconds (under LCF) or 7000 milliseconds (under HCF) post-final stimulus. We adhered to the HRV analysis guidelines outlined by the Task force of the European Society of Cardiology and the North American Society of Pacing and Electrophysiology (Malik, 1996).

## Data Analysis

For each condition, the mean hit rate was calculated as a percentage by taking the total number of a specific type of shot outcome—such as hits toward targets, ironic errors, or non-ironic errors—and dividing it by the total number of incongruent trials, then multiplying the result by 100. Separately, the mean RT was computed by averaging the RTs from correct target hits only.

In the main analysis, we primarily used Bayesian analysis method^5^ with JASP (JASP Team, 2024 v. 0.18.3). The Bayesian method offers a robust framework to effectively address key challenges, such as small sample size and undefined sampling plans (Skorski & Hecksteden, 2021), by incorporating prior evidence and systematically updating it with observed new data to draw strong inferences even from limited datasets (Wagenmakers et al., 2018). We set the Cauchy prior width to default *r* = 0.707. Moreover, we supplemented the analysis with frequentist method using SPSS 28.

In the frequentist analysis, we performed a Pearson correlation to assess the relationship between biathletes’ SAS-N worry subscale scores and their cognitive anxiety (MRF-3) ratings. A one-way repeated measures MANOVA was used to evaluate the effect of conditions (LCF, HCF) on shooting performance (target, ironic error, non-ironic error). A paired samples t-test compared RTs for target hits across conditions. A one-way MANOVA was employed to examine the influence of workload, including perceived exertion, on target shooting performance. The significance level (α) was set at .05. The study design and analyses were not pre-registered.

Two observers independently reviewed video recordings of biathletes’ performance (hits only) to assess interobserver reliability. An intraclass correlation coefficient (ICC) was computed in SPSS 28 according to the guidelines of Koo and Li’s (2016).

## Results

### Trait Anxiety

The results reveal a nonsignificant correlation between SAS-N worry scale and MRF-3 cognitive anxiety under HCF conditions (*r* = .32, *n* = 10, *p* = .37).

### Subjective and Psychophysiological Impact of Instruction Manipulation

Table 1 presents descriptive statistics for subjective and objective measures of instructional manipulation. Mean cognitive anxiety, HR, and HRV did not differ significantly between HCF and LCF conditions, suggesting that frequent priming of negative cues did not deplete biathletes’ attentional resources during the Stroop rifle shooting task.

**Table 1.** Descriptive Statistics for the Manipulation Check

Measure	Condition (*M ± SD)*		t (9)
	LCF	HCF	
Cognitive anxiety	4.50 ± 2.80	4.40 ± 2.37	0.88
Heart rate (beats/min)	88.38 ± 12.77	86.51 ± 12.28	0.12
r-MSSD (ms)^a^	38.21 ± 18.74	39.13 ± 19.99	0.68

^a^The r-MSSD is a measure of HRV that directly reflects the activity of parasympathetic nervous system, also known as “vagal tone” (Laborde et al., 2018).

Abbreviations: LCF = Low-Frequency Cue; HCF = High-Frequency Cue; r-MSSD = root mean square of successive difference between R-R intervals; ms = milliseconds.

### Bayesian Analysis

A Bayesian paired samples t-test was conducted in JASP using a noninformative Cauchy prior (r = .707) and an alternative hypothesis (Measure 1 ≠ Measure 2). The Bayes Factor (BF_01_) was calculated using the Student's t-test. Results showed a BF_01_ of 2.77 < 0.01% for target hit rates, 3.19 < 0.01% for non-ironic error hit rates, and 1.49 < 0.02% for RTs towards target hits. These values indicate that the BF is about 2.8, 3.2, and 1.5 times more likely in favor of the null model (BF_01_) for target, non-ironic error, and RTs towards target hits, respectively, than the alternative model (BF_10_).

A robustness test confirms the stability of the BF even when varying the Cauchy prior width. Based on Lee and Wagenmakers’ (2014), the BF provides moderate evidence for the null hypothesis regarding target and non-ironic error hit rates, and anecdotal evidence for the null hypothesis in target RTs. Refer to Table 2 and Supplemental Figure 2 for a graphical representation of Bayesian analysis.

**Table 2.** JASP Output Table for the Bayesian Paired Samples T-Test for Target Hit Rates and RTs

Measure 1	Measure 2	Bayesian			Frequentist			
		Models			Mean difference			
		BF_01_	BF_10_	Error %	*t*	*df*	*p*	*Cohen’s δ*
TAR_LCF_	TAR_HCF_	2.77	.36	.01	*−.61*	*9*	*.56*	*−.19*
NIE_LCF_	NIE_HCF_	3.19	.31	.01	−.17	9	.87	−.05
RT_LCF_	RT_HCF_	1.49	.67	.02		9	.19	−.45

*Note.* (1) Lee and Wagenmakers (2014) provide a classification scheme for interpreting the strength of evidence provided by the Bayes Factor (BF) of the null hypothesis (H_0_). A BF_01_ value of 1 indicates no evidence, as the data are equally supportive of the null and alternative hypotheses. Values between 1 and 3 are considered anecdotal evidence for H_0_, values from 3 to 10 represent moderate evidence, and values from 10 to 30 constitute strong evidence) for H_0_; (2) The “Frequentist” column shows mean differences and *t*-test values for target hit rates and RTs towards target hits under LCF and HCF conditions. The “Bayesian” column lists the models under consideration. “BF_01_” and “BF_10_” columns depict the BF for comparisons with alternative and null models, respectively. “% error” column reveals error size in the integration routine relative to the BF; (3) Abbreviations: TAR = Target; NIE = Non-ironic error; RT = Response times; LCF = Low-Cue Frequency condition; HCF = High-Cue Frequency condition.

### Frequentist Method

A nonsignificant Box’s *M* test (*p* = .12) indicates homogeneity of covariance matrices of the dependent variables across conditions. The one-way multivariate effect was nonsignificant for condition, *F* (2,17) = 1.02, *p =* .38, η^2^p = .12. Univariate analyses revealed a nonsignificant effect on target hits, *F* (1,18) = 1.93, *p =* .18, η^2^p = .09, and non-ironic error hits, *F* (1,18) = .85, *p =* .37, η^2^p = .05, under LCF and HCF conditions. No ironic error hits occurred in any conditions. The results of shooting performance and RTs are summarized in Table 3.

**Table 3.** Mean Hit Rates of Target, Ironic Error and Non-ironic Error, and Mean Shooting RTs of Target Hits at Incongruent Stimuli Across Conditions

Performance	Mean hit rate (%)		Mean shooting RTs (ms)	
	Incongruent [M (SD)]		Incongruent [M (SD)]	
	LCF	HCF	LCF	HCF
Target	91.50 (8.18)	95.75 (5.14)	1364.40 (144.27)	1408.90 (161.19)
Ironic error	0.00 (0.00)	0.00 (0.00)		
Non-ironic error	5.00 (7.07)	2.50 (4.86)		

*Note.* The mean difference in target hits between LCF and HCF was −2.00 (95% CI = [−9.44, 5.44]). For non-ironic error hit, the mean difference was −0.50 (95% CI = [−7.13, 6.13]). The mean difference in RTs for target hits was −44.50 (95% CI = [−115.65, 26.65]). Abbreviations: LCF = Low-Cue Frequency condition; HCF = High-Cue Frequency condition; RTs = Response times; ms = milliseconds; M = Mean; SD = Standard Deviations.

### Shooting RTs

Overall, biathletes’ RTs toward target hits were found to be statistically nonsignificant across conditions, *t(9)* = −1.42, *p* = .19. However, biathletes reacted faster under LCF (*M* = 1364 milliseconds) than under HCF condition (*M* = 1409 milliseconds).

### Workload

The one-way multivariate effect was nonsignificant for condition, *F* (3, 16) = .12, *p =* .95, η^2^p = .02. The univariate test results indicated a nonsignificant effect for mental demand, *F* (1, 18) = .28, *p =* .61, η^2^p = .02, temporal demand, *F* (1, 18) = .00, *p =* .96, η^2^p = .00, and effort, *F* (1, 18) = .04, *p =* .84, η^2^p = .00 across conditions. However, biathletes perceived the task more mentally challenging under HCF (*M* = 318, *SD* = 77.43) than under LCF (*M* = 302, *SD* = 56.92) and exerted greater effort under HCF (*M* = 387.50, *SD* = 90.71) than under LCF (*M* = 380.00, *SD* = 74.35).

### Post-Manipulation Check

Table 4 presents the level of agreement among biathletes’ on social-evaluation statements.

**Table 4.** Descriptive Statistics About the Biathletes’ Response on Social Evaluation Statements

Statements	Frequency and percentage of response				*Mean*	*SD*
	SD	D	A	SA		
The task instructions were clear	0	0	1	9	3.90	.32
I Followed the instructions diligently	0	0	5	5	3.50	.53
Few distractions, internal and external, caught my attention, causing me to lose focus	10	0	0	0	1.00	.00
Unwanted or irrelevant thoughts often popped into my mind	5	2	3	0	1.80	.92
I Used self-control strategies to accomplish the task	0	1	5	4	3.30	.67
The task was boring	10	0	0	0	1.00	.00
The task was interesting	0	0	1	9	3.90	.32
The task was easy	0	6	3	1	2.50	.71

*Note*. Abbreviations: SD = strongly disagree; D = disagree; A = agree; SA = strongly agree.

### Interobserver Reliability

The ICC result showed excellent reliability with a value of 0.99 (95% CI: 0.992–0.995).

## Discussion

This study examined the impact of repetitive negative cues on ironic errors in elite biathletes. Results showed no significant effect on target hits, indicating highly accurate shooting performance under both LCF and HCF conditions. Contrary to expectations, Wegner’s (1994) ironic error hypothesis was not supported, as no hits occurred on ironic error targets. Additionally, negative cues did not significantly affect shooting RTs across both conditions.

## Theoretical Implications

From a theoretical perspective, the operating process, which primarily governs “information processing in adaptive manner” (Janelle, 1999, p. 205), actively searched for cues aligned with the intended goals. In contrast, the monitoring process might facilitate mental and motor control by not interfering with the conscious mind (Wegner, 1994), even under high activation of negative cue conditions. Alternatively, biathletes’ performance can be framed through the lens of attentional control theory (ACT; Eysenck et al., 2007). ACT highlights two key central executive functions: inhibition (blocking distractions or interferences) and shifting (maintaining focus on the target). When these functions work effectively, the goal-directed (top-down) attentional system dominates, while the stimulus-driven (bottom-up) attentional system becomes less influential (Eysenck et al., 2007). This allows athletes to suppress all irrelevant stimuli, including ironic and non-ironic targets. Consequently, they efficiently filter out distractions, enabling focused and high-performing task execution. However, these interpretations should be approached cautiously, as the dual-process models they are built upon are part of a metaphysical, rather than strictly empirical inquiry (Gawronski & Creighton, 2013).

### Considering Expertise

The monitoring process, which supposedly responsible for creating ironic errors, operates based on an individual’s skill and experience (Janelle, 1999). Elite youth biathletes receive extensive shooting training from an early age through their clubs, developing a high level of skill and experience through practice and competition. This is evidenced by the fact that elite biathletes’ shooting performance aligned with their intended direction of mental control, showing no ironic or negligible non-ironic errors in a novel task. Their significant competition experience at national and international levels has further enhanced their cognitive processing efficiency, allowing them to effectively manage multiple demands and improve motor performance (Cheng et al., 2023). This proficiency fosters positive cognitive, behavioral, and emotional patterns, potentially helping biathletes align their motor actions with their intentions in unfamiliar cognitive-motor task.

Furthermore, expert shooters excel at suppressing task-irrelevant information, focusing on relevant cues, reorienting attention as needed, and achieving greater automaticity (Mon-López et al., 2022; Voss et al., 2010). This enhanced control allows them manage distractions more effectively than novices by utilizing more attentional resources (Abernethy et al., 2007).

Biathletes’ shooting performance could be linked to their ability to visually locate targets, even when repeatedly exposed to negative cues. This highlights the potential importance of gaze behavior. Previous studies have connected elite biathletes performance to specific gaze patterns like prolonged aim-point fixation, during shooting tasks (e.g., Janelle et al., 2000; Vickers & Williams, 2007). It is important to note, however, that our study did not directly measure gaze behavior; therefore, this interpretation remains hypothetical. Moreover, their quick response to targets likely stem from automatic shooting skills developed through target-focused practice (Brown et al., 2013) and extensive shooting time training (Laaksonen et al., 2018), enabling them to save time. In biathlon competition, shooting accuracy is critical because missed targets incur time penalties, making speed as important as accuracy on the range (Luchsinger et al., 2019).

However, it is important to recognize that elite youth biathletes’ resilience against negative priming or cognitive interference during a Stroop shooting task does not stem from age or experience alone. Instead, it results from an integrated developmental process. This process is cultivated through the established structure of the Norwegian Elite Sport Academy (NTG Geilo) environment, which systematically balances high academic and performance challenges with strong relational and institutional support. Within this system, biathletes’ actively use trained psychological skills (see Abrahamsen et al., 2024; Josefsson et al., 2021), physiological techniques (see Žák & Ondráček, 2021), and compensatory strategies (see Gallicchio et al., 2016; Hatfield & Hillman, 2001) for pressure regulation, enhancing neurophysiological control and optimizing performance, even under physically demanding conditions. This holistic cultivation enables a key dissociation: it allows their objective performance remain stable even as their subjective anxiety may rise. This pattern is corroborated by a research on youth volleyball players, who maintained performance stability and unchanged HRV when instructed not to serve to a specific spot, even under pressure conditions, despite reporting elevated cognitive and somatic anxiety (Gorgulu & Gokcek, 2021).

## Empirical Findings

### Considering Cue Priming and Timing

Repeatedly using visual negative cues like “do not shoot blue” before incongruent stimuli under HCF conditions could assist biathletes focus on task-relevant cues, improving target shooting accuracy and speed. Particularly, presenting priming cues for 2000 milliseconds before shooting significantly increases their activation level, enabling biathletes in maintaining selective attention on task-related information. In the mainstream psychology, evidence indicates that visual cues can enhance the brain’s ability to absorb and process information (Chandler & Sweller, 1991), leading to “effective informational influence that causes true cognitive change” (Hogg & Vaughan, 2018, pp. 257–258).

Although a descriptive 45 milliseconds delay was observed, this study found no statistically significant evidence that repeated priming negative cues slowed biathletes’ shooting RTs. This contrasts with established research, where negative priming slows responses due to interference from the suppressed information (Frings et al., 2015; Mills et al., 2019). However, the mechanism by which such priming influences motor actions in IPT is still a subject of debate (see Sparrow & Wegner, 2006).

### Considering Psychophysiological Measures

The nonsignificant changes in HRV (specifically r-MSSD) suggest that elite biathletes’ vagal tone remained stable across cue frequency conditions. This psychophysiological stability likely reflects exceptional self-regulation skills (see Laborde et al., 2018). Despite cognitive interference (from Stroop task) when given negative cues, these elite biathletes maintained a resilient parasympathetic state, which allowed them to effectively mobilize attentional resources and achieve high shooting accuracy. However, it is important to note that our finding of nonsignificant HRV challenges the typical pattern observed in studies of ironic performance under pressure in perceptual-motor tasks. Previous research studies (on non-elite populations) show that trying to avoid a specific motor actions increases psychophysiological arousal (as indicated by low HRV metrics) and disrupts motor control, leading to the ironic errors (e.g., Gorgulu, 2019; Woodman et al., 2015).

According to a recent study, elite shooters exhibit superior self-control compared to their sub-elite counterparts (Englert et al., 2021). The key differentiator appears to be biathletes’ trained ability to synchronize every shot with their breathing rhythm—typically a practice of one breath per shot instilled from the beginning of their carriers in rifle shooting (Konttinen, N., & Lyytinen, 1992; Laaksonen et al., 2018). This *focused breathing* not only aids in effectively suppressing distracting thoughts (see also Wegner, 2011, p. 675) but also modulates cardiac activity (specifically HR and HRV; see Laborde et al., 2024 for a review and meta-analysis), thereby potentially enhancing self-regulation and overall shooting performance. However, this interpretation is speculative, as the study’s design did not directly measure how breathing influences cardiac activity or shooting performance.

### Considering Workload

The NASA-TLX workload results indicate that biathletes perceived the task as more mentally demanding under HCF (*M* = 318) than under LCF (*M* = 302), though the difference was not statistically significant. The findings that biathletes perceived the task more time-demanding and exerted greater effort under HCF (*M* = 388 versus LCF *M* = 380) indicates that increased effort could compensate for the absence of ironic errors. The proposed mechanism for this compensation is that increased effort enhances performance by sharpening focus on goal-relevant stimuli (Eysenck & Calvo, 1992; Eysenck et al., 2007; Wegner, 1994; Wilson et al., 2007), reducing cognitive load, minimizing interruptions, preserving mental capacity, and improving perceptual-motor performance (Moran, 2009; Nieuwenhuys & Oudejans, 2012).

### Considering the Task

The study used a Stroop task, a well-established measure of attention (MacLeod, 1992), adapted to examine cognitive-motor interactions in the context of rifle shooting. While biathletes might benefit from quickly matching target and word colors, potentially improving speed and accuracy, the task’s apparent simplicity is misleading. Despite its many variants—limited by ecological validity, interpretation challenges, practice effects, task specificity, and lack of standardization (see Viviani et al., 2023)—we argue that the Stroop task used in this study is not trivial for four key reasons. First, participants may disengage or lack motivation if they perceive the task is unimportant or overly complex (Chen et al., 2023; Low et al., 2022), potentially leading them to conserve resources—even though task difficulty/complexity was not measured in the study. Second, biathletes found the task engaging, thrilling, and challenging, with a few dissenting (see Table 4 for the social evaluation survey results). The NASA-TLX results indicated that the task was mentally demanding, particularly under the HCF condition, requiring significant mental effort. This supports the notion that effortful tasks consume attentional resources (Janelle, 1999), as biathletes found the unfamiliar Stroop task cognitively taxing, demanding sustained attention, and cognitive control. Third, biathletes effectively managed the novel, cognitively demanding task, as evidenced by nonsignificant effects in objective measures, indicating strong cognitive processing efficiency and capacity (Abernethy et al., 2007). This likely contributed to faster responses and consistent performance, indicating superior working memory capacity^6^. However, this interpretation is speculative, as working memory capacity was not directly measured in our study.

### Considering Post-Test Manipulation Check

Social-evaluation survey indicated that all biathletes reported high levels of concentration, which may help maintain focus during critical shooting moments, even under cognitive load and physical strain (Josefsson et al., 2021). Despite this, 2.5% of elite biathletes’ shot landed on non-ironic error targets under HCF condition, suggesting momentary lapses in attention to task-irrelevant targets. Although trends were nonsignificant and data were analyzed at the group level, these lapses could hold significance, particularly in critical moments.

## Study Limitations, Methodological Considerations, and Future Directions

The Priori was not feasible to determine sample size because the recruitment relied on voluntary sampling. However, we conducted a sensitivity power analysis using G*Power (Faul et al., 2007). At least 84 more biathletes were required to detect a small-to-medium effect size of 0.28 with a power of 80% and alpha of 0.05 in a t-test (difference between two dependent means of RTs) under LCF and HCF conditions. Although large samples are inherently challenging to obtain in elite population (Koch & Krenn, 2021), constraints like time and access further limited our recruitment. Consequently, the number of available biathletes at NTG, Geilo was smaller than the sample size our study required. While the Bayesian analysis somehow addressed the limitations of this study, particularly the small sample size, its necessity underscores the need for future research to secure larger, more diverse elite samples. This would increase statistical power and reduce the reliance on statistical inferences.

Through a novel, unconventional Stroop rifle shooting task, the biathletes in this study developed two key cognitive skills: selective attention (filtering distractors like ironic and non-ironic) and conflict resolution (suppressing an automatic response to execute a controlled one). These skills are analogous to the focused (suppressing unwanted thoughts), adaptive thinking required in high-pressure competitions. Certain limitations of the task, however, should be noted. Unlike the traditional Stroop test (Stroop, 1935), which requires lengthy trials over hours, our shooting task prioritized ecological validity, with biathletes completing 20 stimuli per condition in under eight-minutes. While a 15-m shooting distance may help biathletes’ targets accuracy and speed, logistical constraints prevented testing at the standard 50-m competition distance. Consequently, to enhance ecological validity, future research should examine the occurrence of ironic errors at regulation distances. To further replicate and improve upon this study, future experimental designs should implement two key modifications: first, arranging Stroop task targets horizontally adjacent to each other to mimic the actual configuration in biathlon, and second, incorporating a fifth, neutral-colored target (e.g., purple) that does not conflict with the standard Stroop color words (e.g., red, green, yellow, blue). This addition would establish a more robust control condition devoid of semantic interference.

Although the frequency of visual activation of cues influences biathletes’ shooting performance, it was not taken as a factor in the analysis of this study. This decision was driven by the specific focus on the conceptualization of ironic errors, which only apply to incongruent stimuli. To extend this work, future research should incorporate different instructional valences (ignore, attend, or control) and modalities (verbal, visual), and investigate a range of cue frequencies (with no-frequency, cues before [at set intervals]). This approach would offer a deeper understanding of frequent activation of cues-ironic errors link and a more robust experimental design. In light of this, future studies on ironic processing in motor actions should treat negative instructions not only as cues, but as emotional stimuli that participants find engaging or personally significant (see Taylor, 1999).

The ineffectiveness of priming negative cue to trigger ironic errors indicates that its effects are moderated by factors like expertise and task-specific context. Furthermore, the lack of significant differences in cognitive anxiety and r-MSSD between LCF and HCF may not indicate a failed manipulation, but rather a marker of elite performance. Elite biathletes likely possess such effective self-regulation skills that they remain physiologically and psychologically stable, even under massive pressure conditions (Englert et al., 2021). Therefore, accurately testing their performance requires using robust methods, such as combining negative cues with multiple stressors that mimic real competitive pressure.

To better simulate real-world pressure in lab tests of ironic performance, future research should shift focus from dual-task to triple-task paradigms (Stefani et al., 2022). This can be achieved by incorporating a third stressor, such as a cognitive task (e.g., emotional Stroop; e.g., Lautenbach et al., 2016), a physical-environmental task (e.g., skiing on a treadmill in an environmental chamber), a biomechanical task (Laaksonen et al., 2018), to mimic the complex mental, physical, and environmental demands of actual biathlon competition. This approach could reveal how elite athletes of different ages and skill levels are affected by these combined pressures, potentially triggering the ironic errors that occur when the stakes are high.

Finally, self-report tools, like the workload index and post-experiment survey, are valuable for identifying additional influencing factors when objective measures are unavailable. However, they are susceptible to biases like social desirability or memory bias. Findings from the NASA-TLX and social-evaluation surveys should therefore be interpreted with caution.

## Practical Implications

This pilot study found no evidence to support Wegner’s (1994) hypothesis that thought suppression leads to ironic errors in a Stroop rifle shooting task. Instead, the results suggest that focused thought suppression may serve as an effective mental control strategy (Wegner, 2011), akin to techniques used by elite and Olympic athletes (Gould et al., 1993). Regular practice of thought suppression can help athletes, coaches, and first responders block unwanted thoughts automatically, potentially reducing performance errors (Wegner, 1994). The study’s findings are reigniting key debates in sport and performance psychology by questioning the prevalence of ironic errors among elite athletes and re-examining how elite youth athletes process negative instructions during cognitive-motor dual-task. These insights have direct practical applications. They can inform the design of more effective thought suppression training and cognitive load management programs. Ultimately, this research can help professional practitioners (such as coaches, mental performance consultants, and sport psychologists) develop frameworks to build more resilient developmental pathways for youth athletes and prevent performance-degrading errors (such as ironic errors or choking).

The stable HRV metrics suggests that vagal regulation is a critical component of elite biathlon shooting performance. Consequently, practical training for elite biathletes should not only focus on physical skiing and technical shooting but should also systematically incorporate methods for developing psychological skills and physiological self-regulation. Notably, implementing intervention protocols like HRV biofeedback and breathing can provide biathletes with direct feedback to consciously cultivate and maintain the vagal tone and attentional control linked to peak performance (Gross et al., 2017; Jiménez et al., 2017).

This study highlights the potential for developing cognitive training programs (like Stoop tasks), which show promise in sports (Walton et al., 2018), could also improve suppression of unwanted thoughts. The primary goal is to enhance cognitive (executive) functions through the implementation of mental and motor control training, along with intervention-based research associated with thought suppression and ironic processes in simulated environments, ultimately improving cognitive and motor performance.

## Conclusion

The study used a novel approach to examine how suppressing negative instructions affects cognitive and motor processes, applying IPT. Results revealed that negative instructions, regardless of frequency, did not significantly affect elite biathletes’ Stroop rifle shooting performance. Specifically, Under the HCF condition, the instruction “e.g., do not shoot blue” failed to guide biathletes’ shooting blue targets. While the NASA-TLX and social-evaluation surveys identified factors that may have reduced the manipulation’s effectiveness, more rigorous research methods are needed to validate these findings.

Conducted in a controlled, “stress-free” environment, this study highlights the impact of situational context on biathletes’ ability to regulate unwanted thoughts, suggesting that the absence of physical and anxiety-inducing stressors may have played a role. The findings raise an important question about the interplay between cognitive and motor processes when responding to negative instructions, particularly using IPT. This highlights the need to reassess how instructions—whether informational or emotional, verbal or visual—are designed and delivered in elite sport settings to better understand IPT.

## Supplemental Material

Supplemental Material - The Effects of Priming Negative Cues on Stroop Rifle Shooting Task in Elite Biathletes: A Psychophysiological Pilot Study Supplemental Material for The Effects of Priming Negative Cues on Stroop Rifle Shooting Task in Elite Biathletes: A Psychophysiological Pilot Study by Khelifa Bartura, Henrik Gustafsson, Frank Eirik Abrahamsen in Perceptual and Motor Skills

## Notes

1. Wegner et al. (1993) conceptualized performance outcome variables in the Stroop word-color task as the target (the desired performance), the ironic error (a specific avoidable targe), and the non-ironic error (a neutral or common error unrelated to the target or ironic error). However, Wegner et al.’s (1998) study did not extend this categorization to their golf putting and pendulum stability tasks. Woodman et al. (2015) were the first to apply this distinct measurement approach to sporting performance.

2. Wegner (1994) contended that once suppressed, unwanted thoughts often return, regardless of priming of the very thoughts repetitively. As such, early thought suppression research studies did not prioritize the frequent priming of unwanted thoughts.

3. Based on McKay et al.’s (2022) framework for defining eliteness.

4. However, we customized the stimuli to the biathlon context, considering target dimension, congruent and incongruent stimuli proportion, and word-target counterbalancing.

5. The Bayesian method offers a robust alternative to the frequentist approach, enabling probabilistic inferences from the data. By comparing null and alternative models, it assesses the strength of evidence for or against an effect (van Doorn et al., 2020).

6. This capacity is crucial in determining the link between mental control and the incidence of ironic errors (Wegner, 1994).
