# Supplementary material for: The Effects of Priming Negative Cues on Stroop Rifle Shooting Task in Elite Biathletes: A Psychophysiological Pilot Study
Source: Percept Mot Skills. 2026 Jan 28;133(6):2069–96. doi: 10.1177/00315125261419408 (PMC13724815; doi:10.1177/00315125261419408)
Supplement: Supplementary file 1 [file sj-pdf-1-pms-10.1177_00315125261419408.pdf]

Supplementary Materials

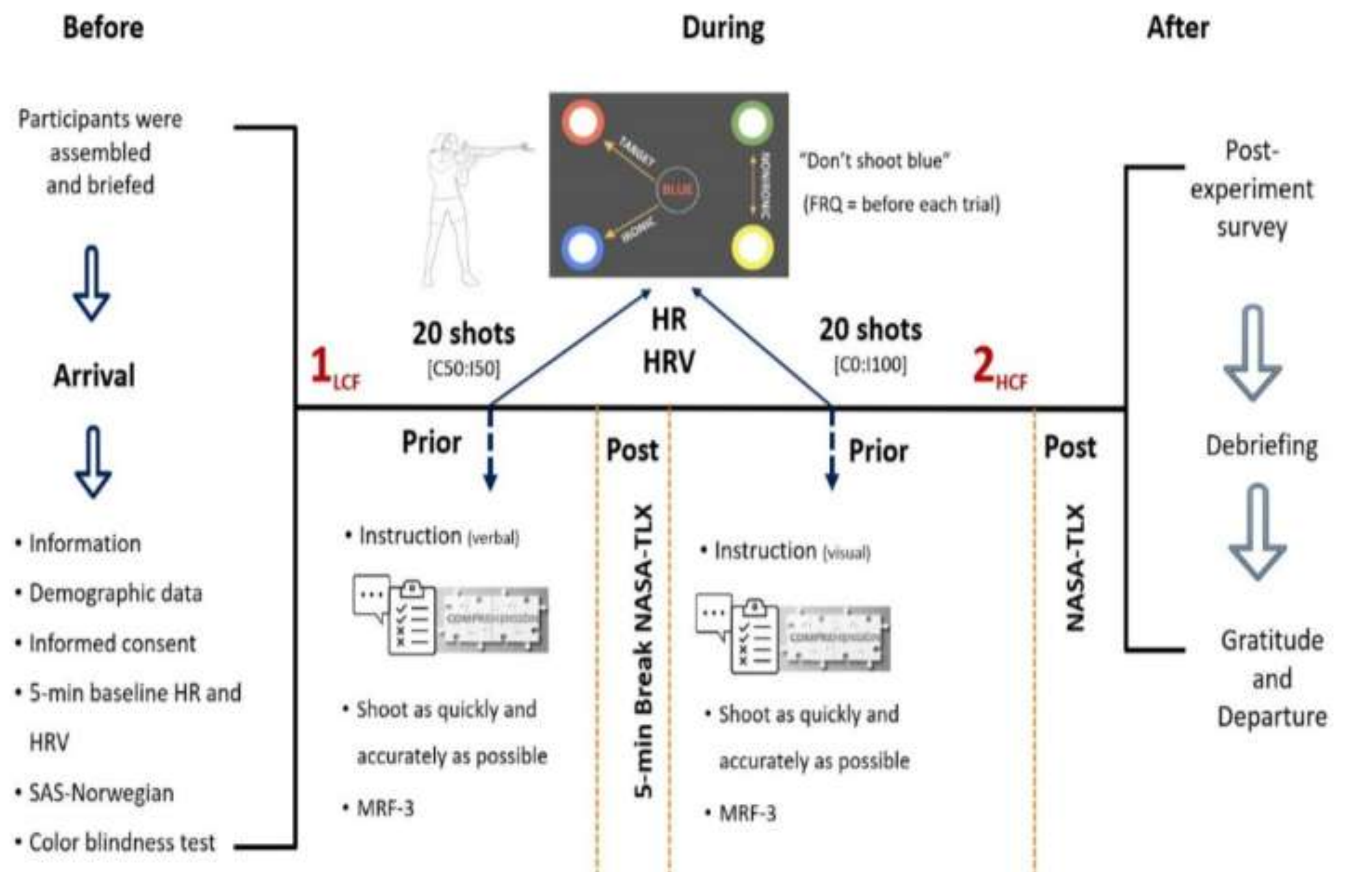

Supplementary Figure 1. Experimental procedure

Note. Abbreviations: HR = Heart Rate; HRV = Heart Rate Variability; SAS = Sport Anxiety Scale; C = Congruent; I = Incongruent; FRQ = Frequency; MRF = Mental Readiness Form; NASA-TLX = National Aeronautics and Space Administration-Task Load Index; 1 = First Trial under Low-Cue Frequency Condition; and 2 = Second Trial under High-Cue Frequency Condition

Supplementary Figure 2. Scoring system of the congruent and incongruent trials

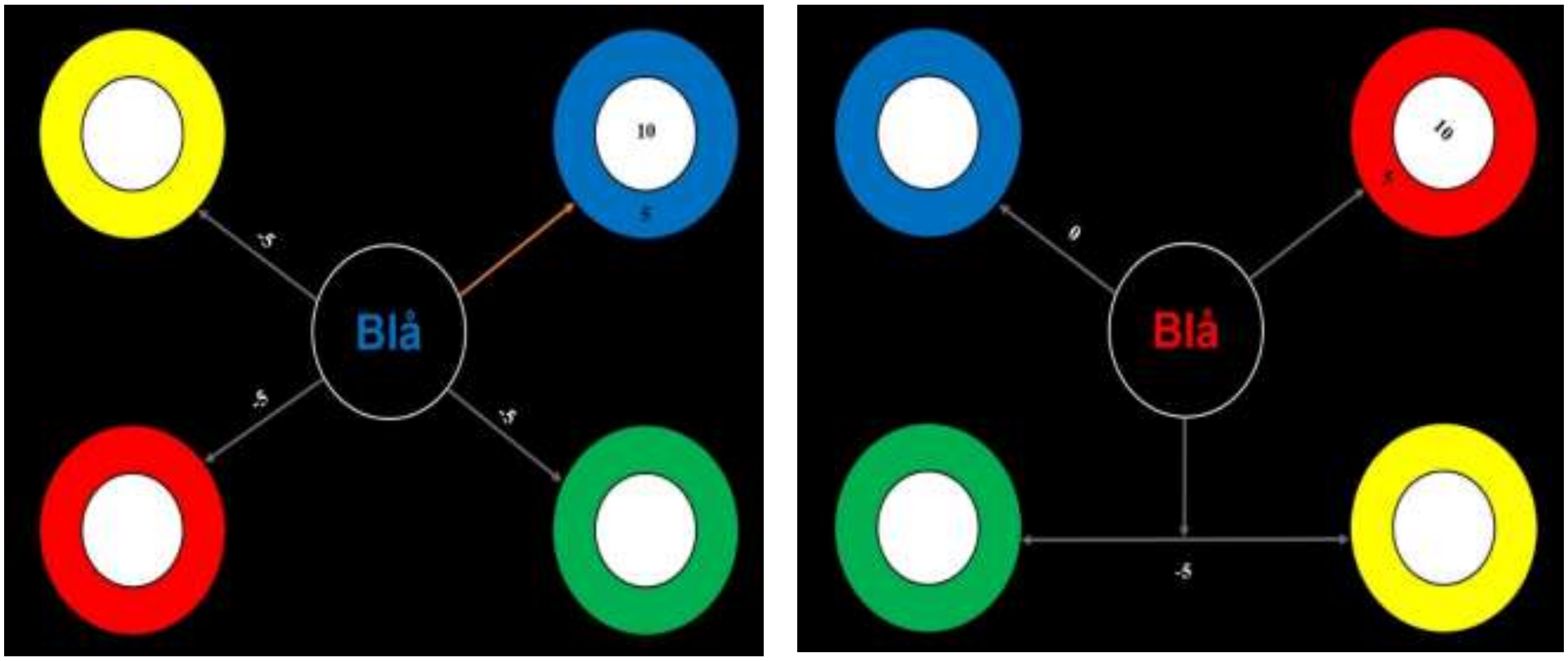

Note. (1) The congruent (Left Panel) and incongruent (Right Panel) stimulus graphical representation for the target shooting task with scoring system; (2) The word Blå in Norwegian = Blue

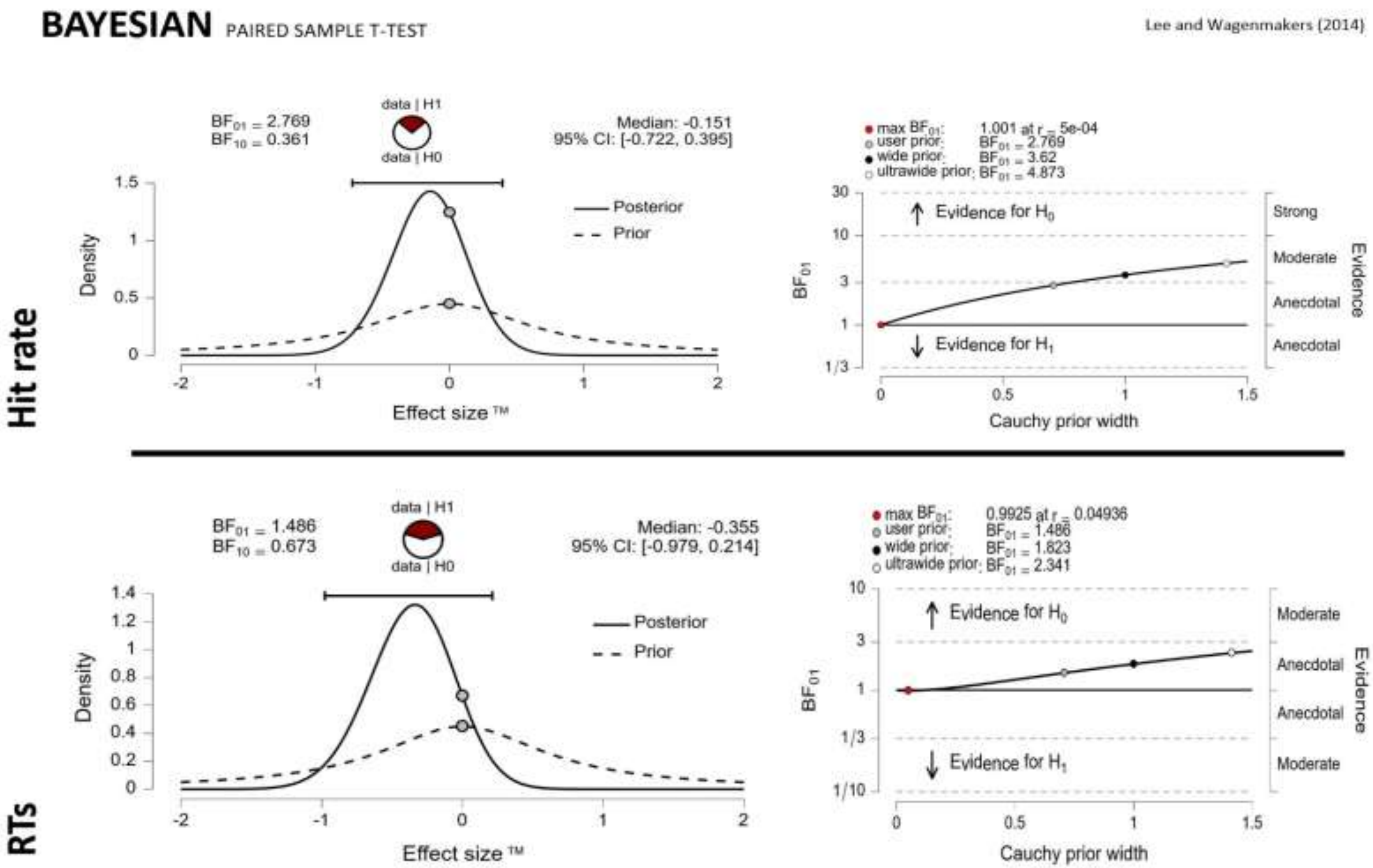

Supplementary Figure 3. Bayes Factor prior, posterior, and robustness test plots for target hit rates and RTs towards target hits

Note. The "BF_{01}" depicts the BF for the comparisons with the alternative model. The "BF_{10}" depicts the BF for the comparisons with the Null model.

Supplementary Table 1. Specifications of apparatus and software used in this study

Type	Model specification / version
**Equipment**	
iPad	Apple iPad 11 Pro (iOS 15, 2020, Apple Inc.
H10 Polar transmitter	Polar Electro Oy, Professorintiet, F1-90440 Kempele, Finland)
Smart phone	Samsung Galaxy S10 Android version 12
Laptop	HP EliteBook 840 G8 Notebook PC
Projector	BenQ MH680; Japan Co. Ltd.
Canvas	A wooden portable frame (190 × 100 cm), accommodating a projected 160 × 90 cm Stroop stimuli[1]
Wireless presenter	Logitech R400
Video camera	4K Panasonic HC-VXF1; 25 fps, 3840 × 2160 p
Portable tripod	Benro KH25N
Rifle	3.5 kilogram, 22 caliber; lead alloy ammo 5.59 millimeters long and 2.55 – 2.75 grams
Earmuffs	Howard Leight L3, 28 Noise Reduction Ratings (NRR)
**Software**	
Camtasia	21.019 TechSmith Corporation
Application	Polar Equine Android version 1.2.1
HRV	Kubios HRV Standard version 3.5.0
Statistics	JASP Team, version 0.19.3
	IBM SPSS Statistics version 28.0.0, IBM Corp

Note. 1. Using the projected image size dimensions (1.6 m wide × 0.9 m high) and a fixed viewing distance of 15 meters, we calculated a visual angel (θ in degrees) subtended by the image. The formula used, based on Palmer's (1999) methodology for visual angle calculation, is: $\boldsymbol{\theta = 2 \times \arctan (s/2d)}$, where s is the size of the object, d is the viewing distance (15 m), arctan is the inverse tangent function. The calculations for the horizontal (θ_h) and vertical (θ_v) visual angles are as follows: $\theta_h = 2 \times \arctan (1.6/2\times15) = 2 \times \arctan (0.05333)$ [arctan 0.05333 ~3.054°], $\theta_h = 2 \times 3.054^o$ ~**6.11°**. Similarly, $\theta_v = 2 \times \arctan (0.9/2\times15) = 2 \times \arctan (0.03)$ [arctan 0.03 ~1.718°], $\theta_v = 2 \times 1.718^o$ ~**3.44°**. Consequently, the projected image subtended a visual angle of approximately 6.11° horizontally and 3.44° vertically. This indicates that the stimulus subtending 6.11° × 3.44° is a medium-sized, foveal-to-parafoveal target that is ideal for ensuring the information is large enough to be clearly visible for biathletes, thereby facilitating easy processing of the Stroop's target color and word.
